# Array programming with NumPy

**DOI:** 10.1038/s41586-020-2649-2

**Published:** 2020-09-16

**Authors:** Charles R. Harris, K. Jarrod Millman, Stéfan J. van der Walt, Ralf Gommers, Pauli Virtanen, David Cournapeau, Eric Wieser, Julian Taylor, Sebastian Berg, Nathaniel J. Smith, Robert Kern, Matti Picus, Stephan Hoyer, Marten H. van Kerkwijk, Matthew Brett, Allan Haldane, Jaime Fernández del Río, Mark Wiebe, Pearu Peterson, Pierre Gérard-Marchant, Kevin Sheppard, Tyler Reddy, Warren Weckesser, Hameer Abbasi, Christoph Gohlke, Travis E. Oliphant

**Affiliations:** 1 Independent researcher, Logan, UT USA; 2 Brain Imaging Center, University of California, Berkeley, Berkeley, CA USA; 3 Division of Biostatistics, University of California, Berkeley, Berkeley, CA USA; 4 Berkeley Institute for Data Science, University of California, Berkeley, Berkeley, CA USA; 5 Applied Mathematics, Stellenbosch University, Stellenbosch, South Africa; 6 Quansight, Austin, TX USA; 7 Department of Physics, University of Jyväskylä, Jyväskylä, Finland; 8 Nanoscience Center, University of Jyväskylä, Jyväskylä, Finland; 9 Mercari JP, Tokyo, Japan; 10 Department of Engineering, University of Cambridge, Cambridge, UK; 11 Independent researcher, Karlsruhe, Germany; 12 Independent researcher, Berkeley, CA USA; 13 Enthought, Austin, TX USA; 14 Google Research, Mountain View, CA USA; 15 Department of Astronomy and Astrophysics, University of Toronto, Toronto, Ontario Canada; 16 School of Psychology, University of Birmingham, Edgbaston, Birmingham UK; 17 Department of Physics, Temple University, Philadelphia, PA USA; 18 Google, Zurich, Switzerland; 19 Department of Physics and Astronomy, The University of British Columbia, Vancouver, British Columbia Canada; 20 Amazon, Seattle, WA USA; 21 Independent researcher, Saue, Estonia; 22 Department of Mechanics and Applied Mathematics, Institute of Cybernetics at Tallinn Technical University, Tallinn, Estonia; 23 Department of Biological and Agricultural Engineering, University of Georgia, Athens, GA USA; 24 France-IX Services, Paris, France; 25 Department of Economics, University of Oxford, Oxford, UK; 26 CCS-7, Los Alamos National Laboratory, Los Alamos, NM USA; 27 Laboratory for Fluorescence Dynamics, Biomedical Engineering Department, University of California, Irvine, Irvine, CA USA

**Keywords:** Computational neuroscience, Solar physics, Computational science, Computer science, Software

## Abstract

Array programming provides a powerful, compact and expressive syntax for accessing, manipulating and operating on data in vectors, matrices and higher-dimensional arrays. NumPy is the primary array programming library for the Python language. It has an essential role in research analysis pipelines in fields as diverse as physics, chemistry, astronomy, geoscience, biology, psychology, materials science, engineering, finance and economics. For example, in astronomy, NumPy was an important part of the software stack used in the discovery of gravitational waves^1^ and in the first imaging of a black hole^2^. Here we review how a few fundamental array concepts lead to a simple and powerful programming paradigm for organizing, exploring and analysing scientific data. NumPy is the foundation upon which the scientific Python ecosystem is constructed. It is so pervasive that several projects, targeting audiences with specialized needs, have developed their own NumPy-like interfaces and array objects. Owing to its central position in the ecosystem, NumPy increasingly acts as an interoperability layer between such array computation libraries and, together with its application programming interface (API), provides a flexible framework to support the next decade of scientific and industrial analysis.

## Abstract (web summary)

NumPy is the primary array programming library for Python; here its fundamental concepts are reviewed and its evolution into a flexible interoperability layer between increasingly specialized computational libraries is discussed.

## Main

Two Python array packages existed before NumPy. The Numeric package was developed in the mid-1990s and provided array objects and array-aware functions in Python. It was written in C and linked to standard fast implementations of linear algebra^3,4^. One of its earliest uses was to steer C++ applications for inertial confinement fusion research at Lawrence Livermore National Laboratory^5^. To handle large astronomical images coming from the Hubble Space Telescope, a reimplementation of Numeric, called Numarray, added support for structured arrays, flexible indexing, memory mapping, byte-order variants, more efficient memory use, flexible IEEE 754-standard error-handling capabilities, and better type-casting rules^6^. Although Numarray was highly compatible with Numeric, the two packages had enough differences that it divided the community; however, in 2005 NumPy emerged as a ‘best of both worlds’ unification^7^—combining the features of Numarray with the small-array performance of Numeric and its rich C API.

Now, 15 years later, NumPy underpins almost every Python library that does scientific or numerical computation^8–11^, including SciPy^12^, Matplotlib^13^, pandas^14^, scikit-learn^15^ and scikit-image^16^. NumPy is a community-developed, open-source library, which provides a multidimensional Python array object along with array-aware functions that operate on it. Because of its inherent simplicity, the NumPy array is the de facto exchange format for array data in Python.

NumPy operates on in-memory arrays using the central processing unit (CPU). To utilize modern, specialized storage and hardware, there has been a recent proliferation of Python array packages. Unlike with the Numarray–Numeric divide, it is now much harder for these new libraries to fracture the user community—given how much work is already built on top of NumPy. However, to provide the community with access to new and exploratory technologies, NumPy is transitioning into a central coordinating mechanism that specifies a well defined array programming API and dispatches it, as appropriate, to specialized array implementations.

## NumPy arrays

**Fig. 1:**
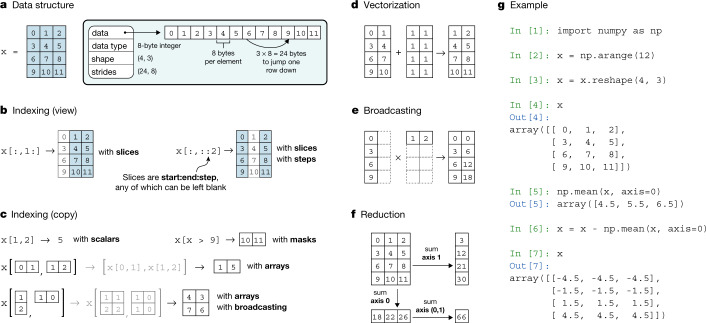
The NumPy array incorporates several fundamental array concepts. **a**, The NumPy array data structure and its associated metadata fields. **b**, Indexing an array with slices and steps. These operations return a ‘view’ of the original data. **c**, Indexing an array with masks, scalar coordinates or other arrays, so that it returns a ‘copy’ of the original data. In the bottom example, an array is indexed with other arrays; this broadcasts the indexing arguments before performing the lookup. **d**, Vectorization efficiently applies operations to groups of elements. **e**, Broadcasting in the multiplication of two-dimensional arrays. **f**, Reduction operations act along one or more axes. In this example, an array is summed along select axes to produce a vector, or along two axes consecutively to produce a scalar. **g**, Example NumPy code, illustrating some of these concepts.

The NumPy array is a data structure that efficiently stores and accesses multidimensional arrays^17^ (also known as tensors), and enables a wide variety of scientific computation. It consists of a pointer to memory, along with metadata used to interpret the data stored there, notably ‘data type’, ‘shape’ and ‘strides’ (Fig. 1a).

The data type describes the nature of elements stored in an array. An array has a single data type, and each element of an array occupies the same number of bytes in memory. Examples of data types include real and complex numbers (of lower and higher precision), strings, timestamps and pointers to Python objects.

The shape of an array determines the number of elements along each axis, and the number of axes is the dimensionality of the array. For example, a vector of numbers can be stored as a one-dimensional array of shape *N*, whereas colour videos are four-dimensional arrays of shape (*T*, *M*, *N*, 3).

Strides are necessary to interpret computer memory, which stores elements linearly, as multidimensional arrays. They describe the number of bytes to move forward in memory to jump from row to row, column to column, and so forth. Consider, for example, a two-dimensional array of floating-point numbers with shape (4, 3), where each element occupies 8 bytes in memory. To move between consecutive columns, we need to jump forward 8 bytes in memory, and to access the next row, 3 × 8 = 24 bytes. The strides of that array are therefore (24, 8). NumPy can store arrays in either C or Fortran memory order, iterating first over either rows or columns. This allows external libraries written in those languages to access NumPy array data in memory directly.

Users interact with NumPy arrays using ‘indexing’ (to access subarrays or individual elements), ‘operators’ (for example, +, − and × for vectorized operations and @ for matrix multiplication), as well as ‘array-aware functions’; together, these provide an easily readable, expressive, high-level API for array programming while NumPy deals with the underlying mechanics of making operations fast.

Indexing an array returns single elements, subarrays or elements that satisfy a specific condition (Fig. 1b). Arrays can even be indexed using other arrays (Fig. 1c). Wherever possible, indexing that retrieves a subarray returns a ‘view’ on the original array such that data are shared between the two arrays. This provides a powerful way to operate on subsets of array data while limiting memory usage.

To complement the array syntax, NumPy includes functions that perform vectorized calculations on arrays, including arithmetic, statistics and trigonometry (Fig. 1d). Vectorization—operating on entire arrays rather than their individual elements—is essential to array programming. This means that operations that would take many tens of lines to express in languages such as C can often be implemented as a single, clear Python expression. This results in concise code and frees users to focus on the details of their analysis, while NumPy handles looping over array elements near-optimally—for example, taking strides into consideration to best utilize the computer’s fast cache memory.

When performing a vectorized operation (such as addition) on two arrays with the same shape, it is clear what should happen. Through ‘broadcasting’ NumPy allows the dimensions to differ, and produces results that appeal to intuition. A trivial example is the addition of a scalar value to an array, but broadcasting also generalizes to more complex examples such as scaling each column of an array or generating a grid of coordinates. In broadcasting, one or both arrays are virtually duplicated (that is, without copying any data in memory), so that the shapes of the operands match (Fig. 1d). Broadcasting is also applied when an array is indexed using arrays of indices (Fig. 1c).

Other array-aware functions, such as sum, mean and maximum, perform element-by-element ‘reductions’, aggregating results across one, multiple or all axes of a single array. For example, summing an *n*-dimensional array over *d* axes results in an array of dimension *n* − *d* (Fig. 1f).

NumPy also includes array-aware functions for creating, reshaping, concatenating and padding arrays; searching, sorting and counting data; and reading and writing files. It provides extensive support for generating pseudorandom numbers, includes an assortment of probability distributions, and performs accelerated linear algebra, using one of several backends such as OpenBLAS^18,19^ or Intel MKL optimized for the CPUs at hand (see Supplementary Methods for more details).

Altogether, the combination of a simple in-memory array representation, a syntax that closely mimics mathematics, and a variety of array-aware utility functions forms a productive and powerfully expressive array programming language.

## Scientific Python ecosystem

Python is an open-source, general-purpose interpreted programming language well suited to standard programming tasks such as cleaning data, interacting with web resources and parsing text. Adding fast array operations and linear algebra enables scientists to do all their work within a single programming language—one that has the advantage of being famously easy to learn and teach, as witnessed by its adoption as a primary learning language in many universities.

Even though NumPy is not part of Python’s standard library, it benefits from a good relationship with the Python developers. Over the years, the Python language has added new features and special syntax so that NumPy would have a more succinct and easier-to-read array notation. However, because it is not part of the standard library, NumPy is able to dictate its own release policies and development patterns.

**Fig. 2:**
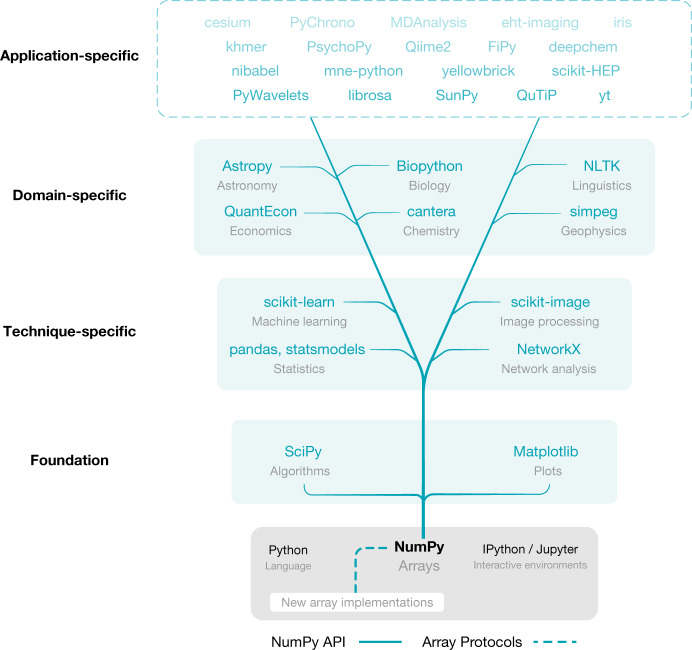
NumPy is the base of the scientific Python ecosystem. Essential libraries and projects that depend on NumPy’s API gain access to new array implementations that support NumPy’s array protocols (Fig. 3).

SciPy and Matplotlib are tightly coupled with NumPy in terms of history, development and use. SciPy provides fundamental algorithms for scientific computing, including mathematical, scientific and engineering routines. Matplotlib generates publication-ready figures and visualizations. The combination of NumPy, SciPy and Matplotlib, together with an advanced interactive environment such as IPython^20^ or Jupyter^21^, provides a solid foundation for array programming in Python. The scientific Python ecosystem (Fig. 2) builds on top of this foundation to provide several, widely used technique-specific libraries^15,16,22^, that in turn underlie numerous domain-specific projects^23–28^. NumPy, at the base of the ecosystem of array-aware libraries, sets documentation standards, provides array testing infrastructure and adds build support for Fortran and other compilers.

Many research groups have designed large, complex scientific libraries that add application-specific functionality to the ecosystem. For example, the eht-imaging library^29^, developed by the Event Horizon Telescope collaboration for radio interferometry imaging, analysis and simulation, relies on many lower-level components of the scientific Python ecosystem. In particular, the EHT collaboration used this library for the first imaging of a black hole. Within eht-imaging, NumPy arrays are used to store and manipulate numerical data at every step in the processing chain: from raw data through calibration and image reconstruction. SciPy supplies tools for general image-processing tasks such as filtering and image alignment, and scikit-image, an image-processing library that extends SciPy, provides higher-level functionality such as edge filters and Hough transforms. The ‘scipy.optimize’ module performs mathematical optimization. NetworkX^22^, a package for complex network analysis, is used to verify image comparison consistency. Astropy^23,24^ handles standard astronomical file formats and computes time–coordinate transformations. Matplotlib is used to visualize data and to generate the final image of the black hole.

The interactive environment created by the array programming foundation and the surrounding ecosystem of tools—inside of IPython or Jupyter—is ideally suited to exploratory data analysis. Users can fluidly inspect, manipulate and visualize their data, and rapidly iterate to refine programming statements. These statements are then stitched together into imperative or functional programs, or notebooks containing both computation and narrative. Scientific computing beyond exploratory work is often done in a text editor or an integrated development environment (IDE) such as Spyder. This rich and productive environment has made Python popular for scientific research.

To complement this facility for exploratory work and rapid prototyping, NumPy has developed a culture of using time-tested software engineering practices to improve collaboration and reduce error^30^. This culture is not only adopted by leaders in the project but also enthusiastically taught to newcomers. The NumPy team was early to adopt distributed revision control and code review to improve collaboration on code, and continuous testing that runs an extensive battery of automated tests for every proposed change to NumPy. The project also has comprehensive, high-quality documentation, integrated with the source code^31–33^.

This culture of using best practices for producing reliable scientific software has been adopted by the ecosystem of libraries that build on NumPy. For example, in a recent award given by the Royal Astronomical Society to Astropy, they state: “The Astropy Project has provided hundreds of junior scientists with experience in professional-standard software development practices including use of version control, unit testing, code review and issue tracking procedures. This is a vital skill set for modern researchers that is often missing from formal university education in physics or astronomy”^34^. Community members explicitly work to address this lack of formal education through courses and workshops^35–37^.

The recent rapid growth of data science, machine learning and artificial intelligence has further and dramatically boosted the scientific use of Python. Examples of its important applications, such as the eht-imaging library, now exist in almost every discipline in the natural and social sciences. These tools have become the primary software environment in many fields. NumPy and its ecosystem are commonly taught in university courses, boot camps and summer schools, and are the focus of community conferences and workshops worldwide. NumPy and its API have become truly ubiquitous.

## Array proliferation and interoperability

NumPy provides in-memory, multidimensional, homogeneously typed (that is, single-pointer and strided) arrays on CPUs. It runs on machines ranging from embedded devices to the world’s largest supercomputers, with performance approaching that of compiled languages. For most its existence, NumPy addressed the vast majority of array computation use cases.

However, scientific datasets now routinely exceed the memory capacity of a single machine and may be stored on multiple machines or in the cloud. In addition, the recent need to accelerate deep-learning and artificial intelligence applications has led to the emergence of specialized accelerator hardware, including graphics processing units (GPUs), tensor processing units (TPUs) and field-programmable gate arrays (FPGAs). Owing to its in-memory data model, NumPy is currently unable to directly utilize such storage and specialized hardware. However, both distributed data and also the parallel execution of GPUs, TPUs and FPGAs map well to the paradigm of array programming: therefore leading to a gap between available modern hardware architectures and the tools necessary to leverage their computational power.

The community’s efforts to fill this gap led to a proliferation of new array implementations. For example, each deep-learning framework created its own arrays; the PyTorch^38^, Tensorflow^39^, Apache MXNet^40^ and JAX arrays all have the capability to run on CPUs and GPUs in a distributed fashion, using lazy evaluation to allow for additional performance optimizations. SciPy and PyData/Sparse both provide sparse arrays, which typically contain few non-zero values and store only those in memory for efficiency. In addition, there are projects that build on NumPy arrays as data containers, and extend its capabilities. Distributed arrays are made possible that way by Dask, and labelled arrays—referring to dimensions of an array by name rather than by index for clarity, compare x[:, 1] versus x.loc[:, 'time']—by xarray^41^.

Such libraries often mimic the NumPy API, because this lowers the barrier to entry for newcomers and provides the wider community with a stable array programming interface. This, in turn, prevents disruptive schisms such as the divergence between Numeric and Numarray. But exploring new ways of working with arrays is experimental by nature and, in fact, several promising libraries (such as Theano and Caffe) have already ceased development. And each time that a user decides to try a new technology, they must change import statements and ensure that the new library implements all the parts of the NumPy API they currently use.

Ideally, operating on specialized arrays using NumPy functions or semantics would simply work, so that users could write code once, and would then benefit from switching between NumPy arrays, GPU arrays, distributed arrays and so forth as appropriate. To support array operations between external array objects, NumPy therefore added the capability to act as a central coordination mechanism with a well specified API (Fig. 2).

**Fig. 3:**
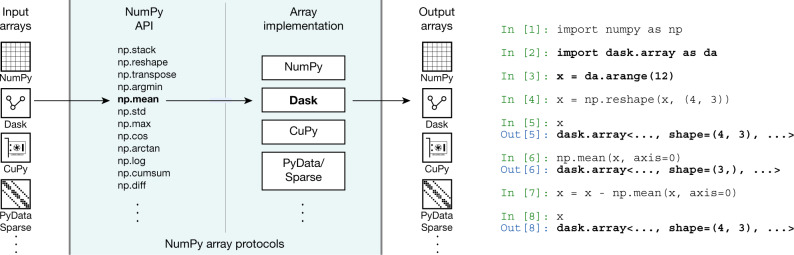
NumPy’s API and array protocols expose new arrays to the ecosystem. In this example, NumPy’s ‘mean’ function is called on a Dask array. The call succeeds by dispatching to the appropriate library implementation (in this case, Dask) and results in a new Dask array. Compare this code to the example code in Fig. 1g.

To facilitate this interoperability, NumPy provides ‘protocols’ (or contracts of operation), that allow for specialized arrays to be passed to NumPy functions (Fig. 3). NumPy, in turn, dispatches operations to the originating library, as required. Over four hundred of the most popular NumPy functions are supported. The protocols are implemented by widely used libraries such as Dask, CuPy, xarray and PyData/Sparse. Thanks to these developments, users can now, for example, scale their computation from a single machine to distributed systems using Dask. The protocols also compose well, allowing users to redeploy NumPy code at scale on distributed, multi-GPU systems via, for instance, CuPy arrays embedded in Dask arrays. Using NumPy’s high-level API, users can leverage highly parallel code execution on multiple systems with millions of cores, all with minimal code changes^42^.

These array protocols are now a key feature of NumPy, and are expected to only increase in importance. The NumPy developers—many of whom are authors of this Review—iteratively refine and add protocol designs to improve utility and simplify adoption.

## Discussion

NumPy combines the expressive power of array programming, the performance of C, and the readability, usability and versatility of Python in a mature, well tested, well documented and community-developed library. Libraries in the scientific Python ecosystem provide fast implementations of most important algorithms. Where extreme optimization is warranted, compiled languages can be used, such as Cython^43^, Numba^44^ and Pythran^45^; these languages extend Python and transparently accelerate bottlenecks. Owing to NumPy’s simple memory model, it is easy to write low-level, hand-optimized code, usually in C or Fortran, to manipulate NumPy arrays and pass them back to Python. Furthermore, using array protocols, it is possible to utilize the full spectrum of specialized hardware acceleration with minimal changes to existing code.

NumPy was initially developed by students, faculty and researchers to provide an advanced, open-source array programming library for Python, which was free to use and unencumbered by license servers and software protection dongles. There was a sense of building something consequential together for the benefit of many others. Participating in such an endeavour, within a welcoming community of like-minded individuals, held a powerful attraction for many early contributors.

These user–developers frequently had to write code from scratch to solve their own or their colleagues’ problems—often in low-level languages that preceded Python, such as Fortran^46^ and C. To them, the advantages of an interactive, high-level array library were evident. The design of this new tool was informed by other powerful interactive programming languages for scientific computing such as Basis^47–50^, Yorick^51^, R^52^ and APL^53^, as well as commercial languages and environments such as IDL (Interactive Data Language) and MATLAB.

What began as an attempt to add an array object to Python became the foundation of a vibrant ecosystem of tools. Now, a large amount of scientific work depends on NumPy being correct, fast and stable. It is no longer a small community project, but core scientific infrastructure.

The developer culture has matured: although initial development was highly informal, NumPy now has a roadmap and a process for proposing and discussing large changes. The project has formal governance structures and is fiscally sponsored by NumFOCUS, a nonprofit that promotes open practices in research, data and scientific computing. Over the past few years, the project attracted its first funded development, sponsored by the Moore and Sloan Foundations, and received an award as part of the Chan Zuckerberg Initiative’s Essentials of Open Source Software programme. With this funding, the project was (and is) able to have sustained focus over multiple months to implement substantial new features and improvements. That said, the development of NumPy still depends heavily on contributions made by graduate students and researchers in their free time (see Supplementary Methods for more details).

NumPy is no longer merely the foundational array library underlying the scientific Python ecosystem, but it has become the standard API for tensor computation and a central coordinating mechanism between array types and technologies in Python. Work continues to expand on and improve these interoperability features.

Over the next decade, NumPy developers will face several challenges. New devices will be developed, and existing specialized hardware will evolve to meet diminishing returns on Moore’s law. There will be more, and a wider variety of, data science practitioners, a large proportion of whom will use NumPy. The scale of scientific data gathering will continue to increase, with the adoption of devices and instruments such as light-sheet microscopes and the Large Synoptic Survey Telescope (LSST)^54^. New generation languages, interpreters and compilers, such as Rust^55^, Julia^56^ and LLVM^57^, will create new concepts and data structures, and determine their viability.

Through the mechanisms described in this Review, NumPy is poised to embrace such a changing landscape, and to continue playing a leading part in interactive scientific computation, although to do so will require sustained funding from government, academia and industry. But, importantly, for NumPy to meet the needs of the next decade of data science, it will also need a new generation of graduate students and community contributors to drive it forward.

## Supplementary information

Supplementary Information This file contains Supplementary Methods, including Supplementary Figure 1 and additional references.
