# Supplementary material for: Array programming with NumPy
Source: Nature. 2020 Sep 16;585(7825):357–62. doi: 10.1038/s41586-020-2649-2 (PMC7759461; doi:10.1038/s41586-020-2649-2)
Supplement: Supplementary file 1 — This file contains Supplementary Methods, including Supplementary Figure 1 and additional references. [file 41586_2020_2649_MOESM1_ESM.pdf]

nature research

https://doi.org/10.1038/s41586-020-2649-2

Supplementary information

Array programming with NumPy

In the format provided by the authors and unedited

Supplementary Methods

Charles R. Harris[1], K. Jarrod Millman[2,3,4,*], Stéfan J. van der Walt[5,2,4,*], Ralf Gommers[6,*], Pauli Virtanen[7,8], David Cournapeau[9], Eric Wieser[10], Julian Taylor[11], Sebastian Berg[4], Nathaniel J. Smith[12], Robert Kern[13], Matti Picus[4], Stephan Hoyer[14], Marten H. van Kerkwijk[15], Matthew Brett[2,16], Allan Haldane[17], Jaime Fernández del Río[18], Mark Wiebe[19,20], Pearu Peterson[6,21,22], Pierre Gérard-Marchant[23,24], Kevin Sheppard[25], Tyler Reddy[26], Warren Weckesser[4], Hameer Abbasi[6], Christoph Gohlke[27], and Travis E. Oliphant[6]

[1]Independent Researcher, Logan, Utah, USA
[2]Brain Imaging Center, University of California, Berkeley, Berkeley, CA, USA
[3]Division of Biostatistics, University of California, Berkeley, Berkeley, CA, USA
[4]Berkeley Institute for Data Science, University of California, Berkeley, Berkeley, CA, USA
[5]Applied Mathematics, Stellenbosch University, Stellenbosch, South Africa
[6]Quansight LLC, Austin, TX, USA
[7]Department of Physics, University of Jyväskylä, Jyväskylä, Finland
[8]Nanoscience Center, University of Jyväskylä, Jyväskylä, Finland
[9]Mercari JP, Tokyo, Japan
[10]Department of Engineering, University of Cambridge, Cambridge, UK
[11]Independent Researcher, Karlsruhe, Germany
[12]Independent Researcher, Berkeley, CA, USA
[13]Enthought, Inc., Austin, TX, USA
[14]Google Research, Mountain View, CA, USA
[15]Department of Astronomy & Astrophysics, University of Toronto, Toronto, ON, Canada
[16]School of Psychology, University of Birmingham, Edgbaston, Birmigham, UK
[17]Department of Physics, Temple University, Philadelphia, PA, USA
[18]Google, Zurich, Switzerland
[19]Department of Physics and Astronomy, The University of British Columbia, Vancouver, BC, Canada
[20]Amazon, Seattle, Washington, USA
[21]Independent Researcher, Saue, Estonia
[22]Department of Mechanics and Applied Mathematics, Institute of Cybernetics at Tallinn Technical University, Tallinn, Estonia
[23]Department of Biological and Agricultural Engineering, University of Georgia, Athens, GA
[24]France-IX Services, Paris, France
[25]Department of Economics, University of Oxford, Oxford, UK
[26]CCS-7, Los Alamos National Laboratory, Los Alamos, NM, USA
[27]Laboratory for Fluorescence Dynamics, Biomedical Engineering Department, University of California, Irvine, Irvine, CA, USA
[*]millman@berkeley.edu, stefanv@berkeley.edu, ralf.gommers@gmail.com

August 12, 2020

We thank the many members of the community who provided feedback, submitted bug reports, made improvements to the documentation, code, or website, promoted NumPy's use in their scientific fields, and built the vast ecosystem of tools and libraries around NumPy. We also gratefully acknowledge the Numeric and Numarray developers on whose work we built.

History

Jim Hugunin wrote Numeric in 1995, while a graduate student at MIT. Hugunin based his package on previous work by Jim Fulton, then working at the US Geological Survey, with input from many others. After he graduated, Paul Dubois at the Lawrence Livermore National Laboratory became the maintainer. Many people contributed to the project including T.E.O. (a co-author of this paper), David Ascher, Tim Peters, and Konrad Hinsen.

In 1998 the Space Telescope Science Institute started using Python and in 2000 began developing a new array package called Numarray, written almost entirely by Jay Todd Miller, starting from a prototype developed by Perry Greenfield. Other contributors included Richard L. White, J. C. Hsu, Jochen Krupper, and Phil Hodge. The Numeric/Numarray split divided the community, yet ultimately pushed progress much further and faster than would otherwise have been possible.

Shortly after Numarray development started, T.E.O. took over maintenance of Numeric. In 2005, he led the effort and did most of the work to unify Numeric and Numarray, and produce the first version of NumPy.

Eric Jones co-founded (along with T.E.O. and P.P.) the SciPy community, gave early feedback on array implementations, and provided funding and travel support to several community members. Numerous people contributed to the creation and growth of the larger SciPy ecosystem, which gives NumPy much of its value. Others injected new energy and ideas by creating experimental array packages.

Version control and collaboration

We use Git for version control and GitHub as the public hosting service for our official *upstream* repository (https://github.com/numpy/numpy). We each work in our own copy (or fork) of the project and use the upstream repository as our integration point. To get new code into the upstream repository, we use GitHub's pull request (PR) mechanism. This allows us to review code before integrating it as well as to run a large number of tests on the modified code to ensure that the changes do not break expected behavior.

We also use GitHub's issue tracking system to collect and triage problems and proposed improvements.

Library organization

Broadly, the NumPy library consists of the following parts: the NumPy array data structure `ndarray`; the so-called *universal functions*; a set of library functions for manipulating arrays and doing scientific computation; infrastructure libraries for unit tests and Python package building; and the program `f2py` for wrapping Fortran code in Python [1]. The `ndarray` and the universal functions are generally considered the core of the library. In the following, we give a brief summary of these components of the library.

Core

The `ndarray` data structure and the universal functions make up the core of NumPy.

The `ndarray` is the data structure at the heart of NumPy. The data structure stores regularly strided homogeneous data types inside a contiguous block memory, allowing for the efficient representation of n-dimensional data. More details about the data structure are given in "The NumPy array: a structure for efficient numerical computation" [2].

The *universal functions*, or more concisely, *ufuncs*, are functions written in C that implement efficient looping over NumPy arrays. An important feature of ufuncs is the built-in implementation of *broadcasting*. For example, the function `arctan2(x, y)` is a ufunc that accepts two values and computes $\tan^{-1}(y/x)$. When arrays are passed in as the arguments, the ufunc will take care of looping over the dimensions of the inputs in such a way that if, say, `x` is a 1-D array with length 3, and `y` is a 2-D array with shape 2×1, the output will be an array with shape 2×3. The ufunc machinery takes care of calling the function with all the appropriate combinations of input array elements to complete the output array. The elementary arithmetic operations of addition, multiplication, etc., are implemented as ufuncs, so that broadcasting also applies to expressions such as `x + y * z`.

Computing libraries

NumPy provides a large library of functions for array manipulation and scientific computing, including functions for: creating, reshaping, concatenating, and padding arrays; searching, sorting and counting data in arrays; computing elementary statistics, such as the mean, median, variance, and standard deviation; file I/O; and more.

NumPy's linear algebra library includes functionality for: solving linear systems of equations; calculating various matrix properties such as the determinant, the norm, the inverse, the pseudo-inverse; and computing the Cholesky, eigenvalue, and singular value decompositions of a matrix.

The NumPy module mainly provides interfaces to established implementations of the LAPACK (Linear Algebra PACKage) [3] interface. LAPACK itself,

which was first released in 1992, is, in turn, based on the EISPACK and LINPACK open-source libraries. It is part of Netlib, a repository of mathematical software, papers, and databases, that has a long history of open, community-wide development [4, 5]. For improved performance, NumPy links to an accelerated BLAS (Basic Linear Algebra Subprograms) implementation, most commonly OpenBLAS (`http://www.openblas.net/`).

The random number generator library in NumPy provides alternative *bit stream generators* that provide the core function of generating random integers. A higher-level generator class that implements an assortment of probability distributions is provided. It includes the beta, gamma and Weibull distributions, the univariate and multivariate normal distributions, and more.

A suite of functions for computing the *fast Fourier transform (FFT)* and its inverse is provided.

Infrastructure libraries

NumPy provides utilities for writing tests and for building Python packages.

The `testing` subpackage provides functions such as `assert_allclose(actual, desired)` that may be used in test suites for code that uses NumPy arrays.

NumPy provides the subpackage `distutils` which includes functions and classes to facilitate configuration, installation, and packaging of libraries depending on NumPy. These can be used, for example, when publishing to the PyPI website.

F2PY

The program `f2py` is a tool for building NumPy-aware Python wrappers of Fortran functions. NumPy itself does not use any Fortran code; F2PY is part of NumPy for historical reasons.

Governance

NumPy adopted an official Governance Document on October 5, 2015 (`https://numpy.org/devdocs/dev/governance/governance.html`). Project decisions are usually made by consensus of interested contributors. This means that, for most decisions, everyone is entrusted with veto power. A Steering Council, currently composed of 12 members, facilitates this process and oversees daily development of the project by contributing code and reviewing contributions from the community.

NumPy's official Code of Conduct was approved on September 1, 2018 (`https://numpy.org/devdocs/dev/conduct/code_of_conduct.html`). In brief, we strive to: *be open*; *be empathetic, welcoming, friendly, and patient*; *be collaborative*; *be inquisitive*; and *be careful in the words that we choose*. The Code of Conduct also specifies how breaches can be reported and outlines the process for responding to such reports.

Funding

In 2017, NumPy received its first large grants totaling 1.3M USD from the Gordon & Betty Moore and the Alfred P. Sloan foundations. Stéfan van der Walt is the PI and manages four programmers working on the project. These two grants focus on addressing the technical debt accrued over the years and on setting in place standards and architecture to encourage more sustainable development.

NumPy received a third grant for 195K USD from the Chan Zuckerberg Initiative at the end of 2019 with Ralf Gommers as the PI. This grant focuses on better serving NumPy's large number of beginning to intermediate level users and on growing the community of NumPy contributors. It will also provide support to OpenBLAS, on which NumPy depends for accelerated linear algebra.

Finally, since May 2019 the project receives a small amount annually from Tidelift, which is used to fund things like documentation and website improvements.

Developers

NumPy is currently maintained by a group of 23 contributors with commit rights to the NumPy code base. Out of these, 17 maintainers were active in 2019, 4 of whom were paid to work on the project full-time. Additionally, there are a few long term developers who contributed and maintain specific parts of NumPy, but are not officially maintainers.

Over the course of its history, NumPy has attracted PRs by 823 contributors. However, its development relies heavily on a small number of active maintainers, who share more than half of the contributions among themselves.

At a release cycle of about every half year, the five recent releases in the years 2018 and 2019 have averaged about 450 PRs each,[1] with each release attracting more than a hundred new contributors. Figure 1 shows the number of PRs merged into the NumPy master branch. Although the number of PRs being merged fluctuates, the plot indicates an increased number of contributions over the past years.

Community calls

The massive number of scientific Python packages that built on NumPy meant that it had an unusually high need for stability. So to guide our development we formalized the feature proposal process, and constructed a development roadmap with extensive input and feedback from the community.

Weekly community calls alternate between triage and higher level discussion. The calls not only involve developers from the community, but provide a venue

[1] Note that before mid 2011, NumPy development did not happen on `github.com`. All data provided here is based on the development which happened through GitHub PRs. In some cases contributions by maintainers may not be categorized as such.

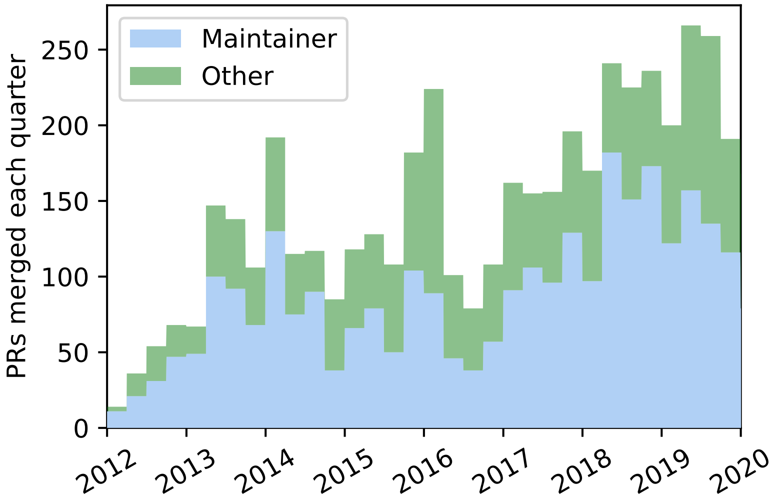

Figure 1: **Number of pull requests merged into the NumPy master branch for each quarter since 2012.** The total number of PRs is indicated, with the lower blue area showing the portion contributed by current or previous maintainers.

for vendors and other external groups to provide input. For example, after Intel produced a forked version of NumPy, one of their developers joined a call to discuss community concerns.

NumPy enhancement proposals

Given the complexity of the codebase and the massive number of projects depending on it, large changes require careful planning and substantial work. NumPy Enhancement Proposals (NEPs) are modeled after Python Enhancement Proposals (PEPs) for "proposing major new features, for collecting community input on an issue, and for documenting the design decisions that have gone into Python"[2]. Since then there have been 19 proposed NEPS—6 have been implemented, 4 have been accepted and are being implemented, 4 are under consideration, 3 have been deferred or superseded, and 2 have been rejected or withdrawn.

[2] https://numpy.org/neps/nep-0000.html

Central role

NumPy plays a central role in building and standardizing much of the scientific Python community infrastructure. NumPy's docstring standard is now widely adopted. We are also now using the NEP system as a way to help coordinate the larger scientific Python community. For example, in NEP 29, we recommend, along with leaders from various other projects, that all projects across the Scientific Python ecosystem adopt a common "time window-based" policy for support of Python and NumPy versions. This standard will simplify downstream project and release planning.

Wheels build system

A Python *wheel* (https://www.python.org/dev/peps/pep-0427/) is a standard file format for distributing Python libraries. In addition to Python code, a wheel may include compiled C extensions and other binary data. This is important, because many libraries, including NumPy, require a C compiler and other build tools to build the software from the source code, making it difficult for many users to install the software on their own. The introduction of wheels to the Python packaging system has made it much easier for users to install precompiled libraries.

A GitHub repository containing scripts to build NumPy wheels has been configured so that a simple commit to the repository triggers an automated build system that creates NumPy wheels for several computer platforms, including Windows, Mac OSX and Linux. The wheels are uploaded to a public server and made available for anyone to use. This system makes it easy for users to install precompiled versions of NumPy on these platforms.

The technology that is used to build the wheels evolves continually. At the time this paper is being written, a key component is the `multibuild` suite of tools developed by Matthew Brett and other developers (https://github.com/matthew-brett/multibuild). Currently, scripts using `multibuild` are written for the continuous integration platforms Travis-CI (for Linux and Mac OSX) and Appveyor (for Windows).

Recent technical improvements

With the recent infusion of funding and a clear process for coordinating with the developer community, we have been able to tackle a number of important large scale changes. We highlight two of those below, as well as changes made to our testing infrastructure to support hardware platforms used in large scale computing.

Array function protocol

A vast number of projects are built on NumPy; these projects are consumers of the NumPy API. Over the last several years, a growing number of projects are providers of a *NumPy-like API* and array objects targeting audiences with specialized needs beyond NumPy's capabilities. For example, the NumPy API is implemented by several popular tensor computation libraries including CuPy[3], JAX[4], and Apache MXNet[5]. PyTorch[6] and Tensorflow[7] provide tensor APIs with NumPy-inspired semantics. It is also implemented in packages that support sparse arrays such as `scipy.sparse` and PyData/Sparse. Another notable example is Dask, a library for parallel computing in Python. Dask adopts the NumPy API and therefore presents a familiar interface to existing NumPy users, while adding powerful abilities to parallelize and distribute tasks.

The multitude of specialized projects creates the difficulty that consumers of these NumPy-like APIs write code specific to a single project and do not support all of the above array providers. This is a burden for users relying on the specialized array-like, since a tool they need may not work for them. It also creates challenges for end-users who need to transition from NumPy to a more specialized array. The growing multitude of specialized projects with NumPy-like APIs threatened to again fracture the scientific Python community.

To address these issues NumPy has the goal of providing the fundamental API for *interoperability* between the various NumPy-like APIs. An earlier step in this direction was the implementation of the `__array_ufunc__` protocol in NumPy 1.13, which enabled interoperability for most mathematical functions (https://numpy.org/neps/nep-0013-ufunc-overrides.html). In 2019 this was expanded more generally with the inclusion of the `__array_function__` protocol into NumPy 1.17. These two protocols allow providers of array objects to be interoperable with the NumPy API: their arrays work correctly with almost all NumPy functions (https://numpy.org/neps/nep-0018-array-function-protocol.html). For the users relying on specialized array projects it means that even though much code is written specifically for NumPy arrays and uses the NumPy API as `import numpy as np`, it can nevertheless work for them. For example, here is how a CuPy GPU array can be passed through NumPy for processing, with all operations being dispatched back to CuPy:

```
import numpy as np
import cupy as cp

x_gpu = cp.array([1, 2, 3])
y = np.sum(x_gpu)  # Returns a GPU array
```

Similarly, user defined functions composed using NumPy can now be applied to, e.g., multi-node distributed Dask arrays:

[3] https://cupy.chainer.org/
[4] https://jax.readthedocs.io/en/latest/jax.numpy.html
[5] https://numpy.mxnet.io/
[6] https://pytorch.org/tutorials/beginner/blitz/tensor_tutorial.html
[7] https://www.tensorflow.org/tutorials/customization/basics

```python
import numpy as np
import dask.array as da


def f(x):
    """Function using NumPy API calls"""
    y = np.tensordot(x, x.T)
    return np.mean(np.log(y + 1))


x_local = np.random.random([10000, 10000])  # random local array
x_distr = da.random.random([10000, 10000])  # random distributed
     array

f(x_local)  # returns a NumPy array
f(x_distr)  # works, returns a Dask array
```

Random number generation

The NumPy `random` module provides pseudorandom numbers from a wide range of distributions. In legacy versions of NumPy, simulated random values are produced by a `RandomState` object that: handles seeding and state initialization; wraps the core pseudorandom number generator based on a Mersenne Twister implementation[8]; interfaces with the underlying code that transforms random bits into variates from other distributions; and supplies a singleton instance exposed in the root of the random module.

The `RandomState` object makes a compatibility guarantee so that a fixed seed and sequence of function calls produce the same set of values. This guarantee has slowed progress since improving the underlying code requires extending the API with additional keyword arguments. This guarantee continues to apply to `RandomState`.

NumPy 1.17 introduced a new API for generating random numbers that use a more flexible structure that can be extended by libraries or end-users. The new API is built using components that separate the steps required to generate random variates. Pseudorandom bits are generated by a bit generator. These bits are then transformed into variates from complex distributions by a generator. Finally, seeding is handled by an object that produces sequences of high-quality initial values.

Bit generators are simple classes that manage the state of an underlying pseudorandom number generator. NumPy ships with four bit generators. The default bit generator is a 64-bit implementation of the Permuted Congruential Generator [6] (`PCG64`). The three other bit generators are a 64-bit version of the Philox generator [7] (`Philox`), Chris Doty-Humphrey's Small Fast Chaotic generator [8] (`SFC64`), and the 32-bit Mersenne Twister [9] (`MT19937`) which has been used in older versions of NumPy.[9] Bit generators provide functions, exposed both in Python and C, for generating random integer and floating point

[8] to be precise, the standard 32-bit version of MT19937

[9] The randomgen project supplies a wide range of alternative bit generators such as a

numbers. The three new bit generators were chosen for their combination of statistical soundness and performance. The PCG family of generators have been widely tested using state-of-the-art statistical tests and found to perform well [11]. All three of the new generators were tested using the PractRand test suite [8] with four TB of random values. Each was tested using a single generator and using a composite generator assembled from multiple copies of the same bit generator, each seeded from a shared `SeedSequence`.

The `Generator` consumes one of the bit generators and produces variates from complicated distributions. Many improved methods for generating random variates from common distributions were implemented, including the Ziggurat method for normal, exponential and gamma variates [12], and Lemire's method for bounded random integer generation [13]. The `Generator` is more similar to the legacy `RandomState`, and its API is substantially the same. The key differences all relate to state management, which has been delegated to the bit generator. The `Generator` does not make the same stream guarantee as the `RandomState` object, and so variates may differ across versions as improved generation algorithms are introduced.[10]

Finally, a `SeedSequence` is used to initialize a bit generator. The seed sequence can be initialized with no arguments, in which case it reads entropy from a system-dependent provider, or with a user-provided seed. The seed sequence then transforms the initial set of entropy into a sequence of high-quality pseudorandom integers, which can be used to initialize multiple bit generators deterministically. The key feature of a seed sequence is that it can be used to spawn child `SeedSequence`s to initialize multiple distinct bit generators. This capability allows a seed sequence to facilitate large distributed applications where the number of workers required is not known. The sequences generated from the same initial entropy and spawns are fully deterministic to ensure reproducibility.

The three components are combined to construct a complete random number generator.

```
from numpy.random import (
    Generator,
    PCG64,
    SeedSequence,
)

seq = SeedSequence(1030424547444117993331016959)
pcg = PCG64(seq)
gen = Generator(pcg)
```

This approach retains access to the seed sequence which can then be used to spawn additional generators.

```
children = seq.spawn(2)
gen_0 = Generator(PCG64(children[0]))
```

cryptographic counter-based generators (`AESCtr`) and generators that expose hardware random number generators (`RDRAND`) [10].

[10] Despite the removal of the compatibility guarantee, simple reproducibility across versions is encouraged, and minor changes that do not produce meaningful performance gains or fix underlying bug are not generally adopted.

```
gen_1 = Generator(PCG64(children[1]))
```

While this approach retains complete flexibility, the method `np.random.default_rng` can be used to instantiate a `Generator` when reproducibility is not needed.

The final goal of the new API is to improve extensibility. `RandomState` is a monolithic object that obscures all of the underlying state and functions. The component architecture is one part of the extensibility improvements. The underlying functions (written in C) which transform the output of a bit generator to other distributions are available for use in CFFI. This allows the same code to be run in both NumPy and dependent that can consume CFFI, e.g., Numba. Both the bit generators and the low-level functions can also be used in C or Cython code.[11]

Testing on multiple architectures

At the time of writing the two fastest supercomputers in the world, Summit and Sierra, both have IBM POWER9 architectures (https://www.top500.org/lists/2019/11/). In late 2018, Astra, the first ARM-based supercomputer to enter the TOP500 list, went into production (https://en.wikichip.org/wiki/supercomputers/astra). Furthermore, over 100 billion ARM processors have been produced as of 2017 (https://en.wikipedia.org/wiki/ARM_architecture), making it the most widely used instruction set architecture in the world.

Clearly there are motivations for a large scientific computing software library to support POWER and ARM architectures. We've extended our continuous integration (CI) testing to include `ppc64le` (POWER8 on Travis CI) and ARMv8 (on Shippable service). We also test with the s390x architecture (IBM Z CPUs on Travis CI) so that we can probe the behavior of our library on a big-endian machine. This satisfies one of the major components of improved CI testing laid out in a version of our roadmap—specifically, "CI for more exotic platforms."

PEP 599 (https://www.python.org/dev/peps/pep-0599/) lays out a plan for new Python binary wheel distribution support, `manylinux2014`, that adds support for a number of architectures supported by the CentOS Alternative Architecture Special Interest Group, including ARMv8, ppc64le, as well as s390x. We are thus well-positioned for a future where provision of binaries on these architectures will be expected for a library at the base of the ecosystem.

References

[1] P. Peterson, "F2PY: a tool for connecting Fortran and Python programs," *International Journal of Computational Science and Engineering*, vol. 4, no. 4, pp. 296–305, 2009.

[11] As of 1.18.0, this scenario requires access to the NumPy source. Alternative approaches that avoid this extra step are being explored.

[2] S. van der Walt, S. C. Colbert, and G. Varoquaux, "The NumPy array: a structure for efficient numerical computation," *Computing in Science & Engineering*, vol. 13, no. 2, pp. 22–30, 2011.

[3] E. Anderson, Z. Bai, C. Bischof, S. Blackford, J. Demmel, J. Dongarra, J. Du Croz, A. Greenbaum, S. Hammarling, A. McKenney, and D. Sorensen, *LAPACK Users' Guide*. Philadelphia, PA: Society for Industrial and Applied Mathematics, 3rd ed., 1999.

[4] J. Dongarra and S. Hammarling, "Evolution of numerical software for dense linear algebra," in *Reliable Numerical Computation* (M. G. Cox and S. Hammarling, eds.), pp. 297–327, Clarendon Press, 1990.

[5] J. Dongarra, G. H. Golub, E. Grosse, C. Moler, and K. Moore, "Netlib and na-net: Building a scientific computing community," *IEEE Annals of the History of Computing*, vol. 30, no. 2, pp. 30–41, 2008.

[6] M. E. O'Neill, "Pcg: A family of simple fast space-efficient statistically good algorithms for random number generation," Tech. Rep. HMC-CS-2014-0905, Harvey Mudd College, Claremont, CA, Sept. 2014.

[7] J. K. Salmon, M. A. Moraes, R. O. Dror, and D. E. Shaw, "Parallel random numbers: As easy as 1, 2, 3," in *Proceedings of 2011 International Conference for High Performance Computing, Networking, Storage and Analysis*, SC '11, (New York, NY, USA), pp. 16:1–16:12, ACM, 2011.

[8] C. Doty-Humphrey, "Practrand, version 0.94." [Online; accessed 1-June-2020].

[9] M. Matsumoto and T. Nishimura, "Mersenne Twister: A 623-dimensionally equidistributed uniform pseudo-random number generator," *ACM Transactions on Modeling and Computer Simulation*, vol. 8, pp. 3–30, Jan. 1998.

[10] K. Sheppard, B. Duvenhage, P. de Buyl, and D. A. Ham, "bashtage/randomgen: Release 1.16.2," Apr. 2019.

[11] D. Lemire, "Testing non-cryptographic random number generators: my results," 2017. [Online; accessed 1-June-2020].

[12] G. Marsaglia and W. W. Tsang, "The ziggurat method for generating random variables," *Journal of Statistical Software, Articles*, vol. 5, no. 8, pp. 1–7, 2000.

[13] D. Lemire, "Fast random integer generation in an interval," *ACM Transactions on Modeling and Computer Simulation*, vol. 29, pp. 1–12, Jan 2019.
